# Reinforcement learning for intensive care medicine: actionable clinical insights from novel approaches to reward shaping and off-policy model evaluation

**DOI:** 10.1186/s40635-024-00614-x

**Published:** 2024-03-25

**Authors:** Luca F. Roggeveen, Ali el Hassouni, Harm-Jan de Grooth, Armand R. J. Girbes, Mark Hoogendoorn, Paul W. G. Elbers, the Dutch ICU Data Sharing Against COVID-19 Collaborators

**Affiliations:** 1 Department of Intensive Care Medicine, Center for Critical Care Computational Intelligence (C4I), Amsterdam Medical Data Science (AMDS), Amsterdam Cardiovascular Science (ACS), Amsterdam Institute for Infection and Immunity (AI&II), Amsterdam Public Health (APH), Amsterdam UMC, Vrije Universiteit, Amsterdam, The Netherlands; 2 Quantitative Data Analytics Group, Department of Computer Science, Faculty of Science, Vrije Universiteit, Amsterdam, The Netherlands

**Keywords:** Reinforcement learning, Explainability, Safety, Mechanical ventilation, Peep, fo2, Covid-19, Cross off-policy evaluation, Policy filtering, Clinical policy inspection

## Abstract

**Background:**

Reinforcement learning (RL) holds great promise for intensive care medicine given the abundant availability of data and frequent sequential decision-making. But despite the emergence of promising algorithms, RL driven bedside clinical decision support is still far from reality. Major challenges include trust and safety. To help address these issues, we introduce cross off-policy evaluation and policy restriction and show how detailed policy analysis may increase clinical interpretability. As an example, we apply these in the setting of RL to optimise ventilator settings in intubated covid-19 patients.

**Methods:**

With data from the Dutch ICU Data Warehouse and using an exhaustive hyperparameter grid search, we identified an optimal set of Dueling Double-Deep Q Network RL models. The state space comprised ventilator, medication, and clinical data. The action space focused on positive end-expiratory pressure (peep) and fraction of inspired oxygen (FiO2) concentration. We used gas exchange indices as interim rewards, and mortality and state duration as final rewards. We designed a novel evaluation method called cross off-policy evaluation (OPE) to assess the efficacy of models under varying weightings between the interim and terminal reward components. In addition, we implemented policy restriction to prevent potentially hazardous model actions. We introduce delta-Q to compare physician versus policy action quality and in-depth policy inspection using visualisations.

**Results:**

We created trajectories for 1118 intensive care unit (ICU) admissions and trained 69,120 models using 8 model architectures with 128 hyperparameter combinations. For each model, policy restrictions were applied. In the first evaluation step, 17,182/138,240 policies had good performance, but cross-OPE revealed suboptimal performance for 44% of those by varying the reward function used for evaluation. Clinical policy inspection facilitated assessment of action decisions for individual patients, including identification of action space regions that may benefit most from optimisation.

**Conclusion:**

Cross-OPE can serve as a robust evaluation framework for safe RL model implementation by identifying policies with good generalisability. Policy restriction helps prevent potentially unsafe model recommendations. Finally, the novel delta-Q metric can be used to operationalise RL models in clinical practice. Our findings offer a promising pathway towards application of RL in intensive care medicine and beyond.

**Supplementary Information:**

The online version contains supplementary material available at 10.1186/s40635-024-00614-x.

## Introduction

The practice of intensive care medicine involves frequent and fast decision-making. To do so, intensive care professionals have access to large amounts of data as intensive care unit (ICU) patients are continuously monitored. Given the often sequential nature of the decision-making process, reinforcement learning (RL) [1] may be a particularly well-suited machine learning method to solve treatment challenges in the ICU. In RL, an agent interacts with an environment, and receives rewards or penalties based on the actions it takes. The agent's goal is to learn a policy that maximises the cumulative reward over time.

RL has enormous potential to improve critical care decision-making by providing a framework for learning from past experience and adapting to changing conditions. RL can be used to model the patient's condition and the potential outcomes of different treatment options, which can aid in the identification of high-risk patients, predict the risk of certain outcomes and find the best treatment pathway for a given patient. However, the majority of these models have been used to generate hypotheses and the use of RL models as bedside decision support aids is still a long way off. Open questions relate to model selection, evaluation metrics, and safe application in a clinical setting [2].

RL models are typically developed and evaluated using a single reward function. A reward function represents the goals of treatment and determines the agent's behaviour in the RL model. However, small changes in the reward function can have profound effects on the developed policies from these trained models [3]. Additionally, the reward signal may differ between patient populations or even individual patients due to operational differences in local guidelines as well as the availability of data used in the reward function. If a model uses a poorly defined reward function, it may generate policies that are not applicable to different patient groups or do not align with varying definitions of successful clinical outcomes.

Further, off-policy evaluation in RL is the process of evaluating the performance of a policy on historical actions with the environment rather than direct interaction of an RL algorithm with patients. Sparse reward signals, such as mortality, is a common challenge in off-policy evaluation as it limits the quality of the evaluation because there may be few or no rewards to base the evaluation on. This problem can be mitigated by adding a more frequently occurring signal to the reward such as incremental improvements of intermediary treatment goals such as restoration of organ function. This adds a new level of complexity as the different components of the reward function must now be weighed against each other. Additionally, if the action recommended by the model only occurs infrequently the quality of the off-policy evaluation degrades severely and can suffer from extreme bias and variance.

Lastly, RL algorithms can be difficult for healthcare providers to interpret and understand, reducing their acceptability. Current research into RL in the ICU mainly focuses on developing policies that are able to achieve better outcomes in terms of patient survival. However, RL can also be used as an analysis tool to evaluate the performance of past treatment strategies and gain insights into which strategies were most effective. This can be particularly useful for identifying patterns in patient care that are associated with better outcomes.

To address these challenges, particularly in regard to safe RL model deployment, we introduce the concept of cross off-policy evaluation. This is based on reward shaping, typically involves altering the reward function to guide the agent towards the desired outcome. Expanding on this, our approach evaluates models based on their performance under different weightings of reward components within a singular reward function. The premise being that a truly robust policy should exhibit its applicability across various reward structures. This ability to generalise is not just an indication of a model's robustness, but also a critical prerequisite for safe model deployment, ensuring that the model remains effective even when circumstances or priorities shift. We not only highlight the potential for this method to identify models that do not generalise effectively, but also showcase how it can be used to identify models that generalise well through the process of elimination. This provides a framework for model evaluation when the ideal reward is difficult to capture in a single number.

We also introduce a method of policy restriction that uses historical actions and the ordinal nature of the action space. This approach helps ensure that recommended actions align with previous treatment steps, thus promoting consistency in policy behaviour. In so doing, the strategy helps to mitigate the risk of unpredictable policy actions, enhancing the reliability of model deployment and contributing to a safer model operation.

Finally, we present different types of policy analysis and show how they can be applied to improve the interpretability from a clinical perspective. We illustrate our proposed solutions by applying them to the development and evaluation of RL models to optimise mechanical ventilation settings for critically ill patients with severe COVID-19 from the Dutch ICU Data Warehouse (DDW).

## Methods

### Clinical context

In this paper we focused on COVID-19 patients that required mechanical ventilation in the ICU to maintain adequate oxygenation and decarboxylation. Important ventilator parameters include respiratory rate, tidal volume, peak pressure, plateau pressure, positive end- expiratory pressure (PEEP) and fraction of inspired oxygen (FiO2). For most of these parameters, the ventilator mode determines whether they are controlled by the healthcare, controlled by the patient or only monitored. However, regardless of ventilator mode, PEEP and FiO2 are always controlled by healthcare professionals rather than only monitored. There is no consensus on optimal values for PEEP and FiO2 [4, 5]. If PEEP is too low, the lung may collapse causing decreased compliance and hypoxemia due to shunting. Overzealous application of PEEP may lead to reduced preload and hence reduced cardiac output, decreased compliance, increased dead-space ventilation causing hypercarbia and acidosis [6]. Similarly, if FiO2 is too low, hypoxia will ensue which will ultimately lead to organ failure and death. However, an FIO2 that is too high is associated with oxygen toxicity to the long and other organs [7]. Personalisation of PEEP and FiO2 may therefore be a valuable strategy. Some recent clinical trials in this direction have shown promising results [8, 9]. However, their approaches are labour intensive and require particular expertise in respiratory physiology as well as additional monitoring devices. Therefore, RL should be promising in this setting. To the best of our knowledge, no previous attempts have been made to use RL to optimise mechanical ventilation settings in COVID-19. There have been a limited number of attempts to use RL for optimising ventilator settings in patients with respiratory failure for reasons other than COVID-19. Prasad et al.'s application of Fitted Q-iteration focuses on optimising weaning protocols in ICUs, with the clinical goal of reducing reintubation occurrences and regulating patient physiological stability [10]. Peine et al. developed a RL-based model called VentAI to minimise 90-day mortality by optimising ventilatory settings, specifically tidal volume (TV), PEEP and FiO2, which are among the most commonly used settings on a ventilator under controlled ventilation [11]. Kondrup et al. proposed DeepVent, a Conservative Q-Learning algorithm (CQL), using a similar setup to Peine et al. and evaluated using Fitted Q Evaluation (FQE) [12]. Additionally, they introduced an intermediate reward function component based on the modified Apache II score.

### Data extraction and preprocessing

**Fig. 1:**
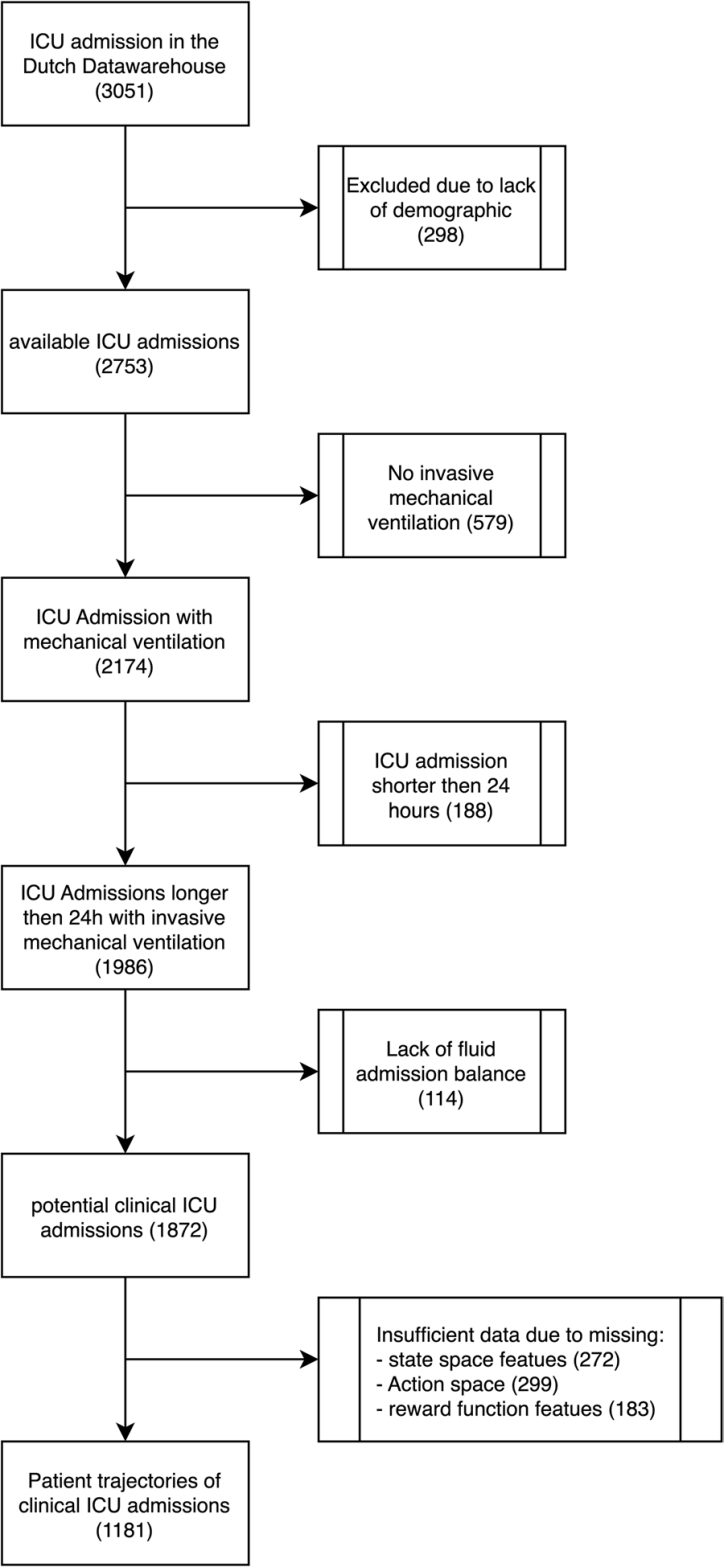
Patient selection flowchart

The data for this study were sourced from the DDW, a database compiling information on critically ill COVID-19 patients from 25 ICUs in the Netherlands [13]. The DDW encompasses data on 3464 patients, covering two distinct periods of the pandemic in the Netherlands, often referred to as "wave 1" and "wave 2". These terms denote the first and the second major surge of COVID-19 cases, respectively, each of which saw a dramatic increase in infections. This database boasts more than 200 million individual clinical data points. At the time of this study, a snapshot of the DWWH, containing 3051 patients rather than the full 3464 were used for this experiment. The overall ICU mortality was 24.4%. Respiratory and haemodynamic parameters were among the most commonly recorded, including ventilation mode, prone position and ventilator settings. Medications administration and daily fluid balance were available for most patients. Lab records were widely available. Clinical features were derived using time aggregations applied at 4, 6, h, 24 h time intervals. A list of all available data parameters and features is available in Additional file 1: Appendix SA. Patient admissions were selected based on length of ICU stay, use of invasive mechanical ventilation, and data availability, for reasons described in Fig. 1.

### Reinforcement learning problem definition

RL is a computational approach where a computer program, known as an 'agent', learns to make decisions by interacting with an environment, in this case, represented by ICU patient data. The goal of the agent is to maximise a 'reward', which in the ICU context, translates to optimal patient treatment outcomes. RL involves the agent interacting with the environment through 'states', which are snapshots of aggregated patient data over specific time intervals, and 'actions', the medical interventions or treatment decisions. A 'trajectory' pertains to the sequential events and decisions made throughout the entire course of a single patient's admission, encompassing the complete set of states and the corresponding actions executed during that individual's stay in the ICU.

Q-learning, an off-policy algorithm in RL, learns optimal decision strategies even from suboptimal actions. In the ICU, this means it can learn effective treatment strategies by analysing both optimal and non-optimal medical decisions made by healthcare professionals. This ability is crucial as it allows for learning from a wide range of historical real-world ICU scenarios without the need for experimental interventions on patients. In Q-learning, a function known as Q(s, a) estimates the utility of taking a particular action (a) in a given state (s), and then following the optimal strategy thereafter.

This section outlines the specific components of our model: the state space, action space, and reward structure, integral to our RL approach.

The state space defines the range of all possible conditions that an ICU patient can experience, as represented by a comprehensive combination of key clinical indicators and measurements. We used one-hour time steps and combined ventilator sensor data, medication records, clinical scores, and laboratory results into one state space. Missing values were imputed using the carry-forward method, up to clinical cut-offs; see Additional file 1: Appendix SA for the full list of features.

**Fig. 2:**
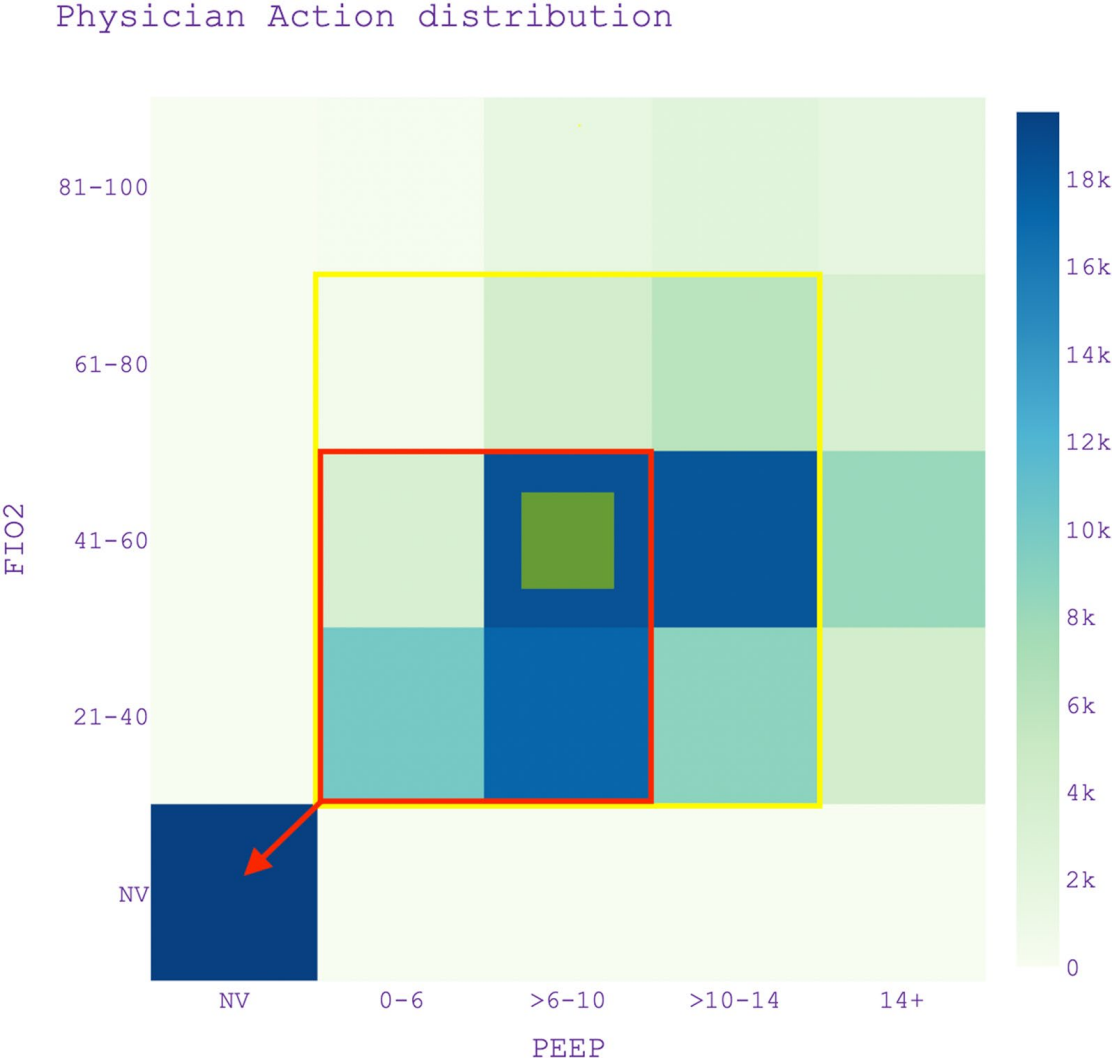
Action space density distribution of the historical actions of physicians in the dataset and illustration of the RL king-knight policy restriction. The red box shows under which actions the RL policy may recommend cessation of mechanical ventilation and the yellow box shows what actions a policy may next recommend if the current action (the small green box) is PEEP 6–10 cmH2O with FiO2 40–60%. NV stands for Non-invasively ventilated

The action space defines the set of all possible interventions and treatment adjustments that the RL model can select from, specifically centred around the settings of positive end-expiratory pressure (PEEP) and fraction of inspired oxygen (FiO2). Figure 2 shows the distribution of actions in the dataset. We used bins of PEEP and FiO2 based on clinical cut-offs, and specified one action for non-ventilation (NV) to allow for the entire ICU admission to be used as a trajectory for training RL models. Our decision to focus the action space on PEEP and FiO2, diverging from the broader dimensions of previous studies [peine `22] [Prassad `17], is strategic. It makes the RL model simpler, reducing the need for extensive training data and computational resources. Concentrating on these key ventilatory parameters allows for direct control, facilitating more straightforward and broadly applicable decision-making. It is worth noting that factors like tidal volume, ventilation mode, and others are incorporated into the state space, ensuring that the model still considers their influence while keeping the action space concise. This approach guarantees the model's adaptability to diverse ICU conditions while maintaining its capacity to provide clear, actionable guidance essential for effective patient care.

In the context of our RL approach, a "reward" serves as a quantitative measure of the quality of patient care, with multiple rewards provided throughout a single patient trajectory. In this study, the reward function encompasses various short-term treatment goals, including oxygenation and ventilation as well as long-term treatment goals such as mortality, length of stay, and discharge destination, collectively guiding the agent towards optimising patient treatment outcomes. For the intermediate reward, we included the P/F-ratio, the ratio of arterial partial pressure of oxygen (PaO2) to inspired oxygen concentration (FiO2), as a measure of oxygenation [14, 15]. The PF-ratio is also used to classify the severity of ARDS [16] and is confirmed as a risk factor for mortality in COVID-19 patients [17]. However, optimal P/F targets are not well-defined [18, 19]. We also included dead space, using Enghoff's modification of Bohr's equation [20] to estimate dead-space ventilation [18] from partial pressure of carbon dioxide (PaCO2) and the end-tidal carbon dioxide (ETCO2) levels: Vd/Vt = [PaCO2 – ETCO2] / PaCO2. Removal of CO2 is a primary goal of mechanical ventilation [21] and dead space is correlated with mortality in ARDS patients [22–25]. As exact targets are ill-defined, we primarily used changes between measurements as a delta target in the intermediate reward formulation. We defined a terminal reward component based on several factors. First, we included mortality, but also included the length of stay and the patient's discharge destination after the hospital admission. The primary objective is to improve the quality of life, which is conventionally measured using Quality-Adjusted Life Years [26]. However, due to insufficient post-discharge data, we employed the length of stay (LOS) in the ICU as a surrogate measure for quality of life. This choice was made to approximate the impact of healthcare interventions on patients' well-being. The exact formula for the reward function is provided in Additional file 2: Appendix SB.

### Policy formulation

In RL models, while the primary goal is to learn optimal policies, these models inherently do not prescribe a specific action as optimal. The common practice is to employ a "greedy" policy, selecting actions with the highest expected reward. However, this approach might not always be suitable, particularly for unstable ARDS patients, where a significant deviation from previous actions could be detrimental. To mitigate this issue, we propose a policy restriction that confines the model to similar actions, allowing for one step up or down in either PEEP or FiO2. Illustrated in Fig. 2, our approach restricts the agent like a chess piece, with movements similar to a chessboard. The agent's deviations are akin to a king's movements, and the RL policy's advice on stopping mechanical ventilation is likewise constrained. We term this approach the "king-knight" policy that allows for structured flexibility, particularly when a patient is not on mechanical ventilation, where any action is permissible.

### Model architecture, training and off-policy evaluation

The model architecture used in this paper is a Dueling Double-Deep Q Network (DDQN) [27, 28] as used in previous work [29]. We used an extensive hyperparameter grid to find a set of optimal model and training settings, including a variable amount of hidden layers (3 to 5) and nodes (32, 64, 128) per layer. Two learning rate (LR) decay (ReduceLRonPlateau and STEPLR) were explored for training. Training was performed using Prioritised Experience Replay [30] and all models were implemented with PyTorch [31]. We used the MAGIC [32]OPE estimator to assess policy performance. We defined the physician behavioural policy using K-nearest neighbour as in previous work [29].

### Experimental setup

**Fig. 3:**
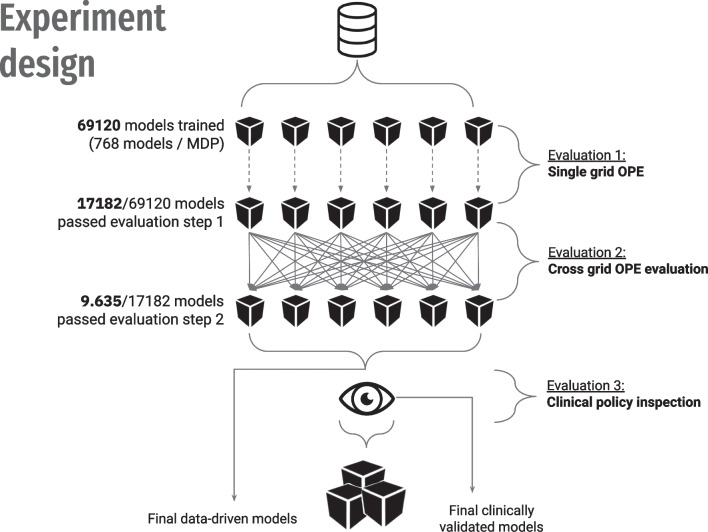
Experiment design with a framework for off-policy evaluation and model and policy selection through cross-OPE evaluation and clinical policy inspection

Given the unknown optional trade-off between intermediate and terminal rewards, our study involved training models using a diverse range of six weightings encompassing both reward components. To assess the generalisability of policies, we introduced cross off-policy evaluation, where policies trained under a specific set of reward weights were evaluated on the remaining five sets of reward weights. In our experiments, this weighting factor is varied across a set of predefined values: [0.25, 0.5, 1, 2, 4, 8]. This evaluation methodology necessitates that each individual reward component, namely the intermediate and terminal reward, inherently reflects clinically desirable outcomes in isolation. The experimental setup and methodology employed are depicted in Fig. 3. Best performing models were selected for further clinical policy inspection.

### Policy evaluation and clinical policy inspection

To assess RL policies, the Off-Policy Policy Evaluation (OPE) method is utilised. OPE allows for the appraisal of a proposed AI policy by estimating its performance using real-world, historical ICU data, thus measuring its potential impact and effectiveness in past patient care scenarios without the need for actual policy execution.

To evaluate policies in a rigorous manner, we propose the introduction of a novel metric termed "delta-Q". Firstly, the Q-value, or q(s,a), represents the output of our model for a given state-action pair, indicating the expected utility of an action in a specific state. Delta-Q, on the other hand, is defined as the difference between the Q-value generated by our model and the Q-value representing the physician's action. Essentially, it measures the discrepancy in action quality between the AI's decision and the physician's choice. A delta-Q of zero implies that the model's decision aligns with the physician's, while a positive delta-Q suggests that the model's action might lead to improved treatment outcomes compared to historical decisions. By examining delta-Q values across all state-action pairs, we can identify specific areas of treatment that warrant further clinical scrutiny. Notably, delta-Q can be employed at both the dataset level and individual patient trajectories, enabling comprehensive analysis.

We also extend upon previous work on clinical policy inspection [29] and propose several model and policy visualisations for clinical validation and operationalisation. Due to the black-box nature of deep learning algorithms, we aim to provide clinical insight into policy behaviour for individual patients. For example, we can use delta-Q as a metric for alignment between physician and policy actions. Sudden changes in delta-Q, due to changes in state or physician action, over the course of the admissions can be used as a clinical alert. This allows physicians to evaluate model behaviour on a case-by-case basis and align with the clinical context in which the trained model and policy will be used.

## Results

We extracted 1118 ICU admission from the DDW to create trajectories. 70% of trajectories were used for training and 30% for testing. The average duration of an admission was 12.8 days, resulting in a total of 34 years of data for training and 7.8 years for testing. Missing values were filled using the last value carry-forward method [33] and patients with excessive missing data were excluded, as shown in Fig. 1. A total of 158 features were used in the final state space. We trained 69,120 models using 8 model architectures and explored 18 hyperparameters. The total training time for this experiment was approximately 7 days on a virtual machine with 32 cores, 32 GB RAM, and 2 Nvidia 3070 Ti GPUs. Training was done using cuda [34].

### Model and policy performance

**Fig. 4:**
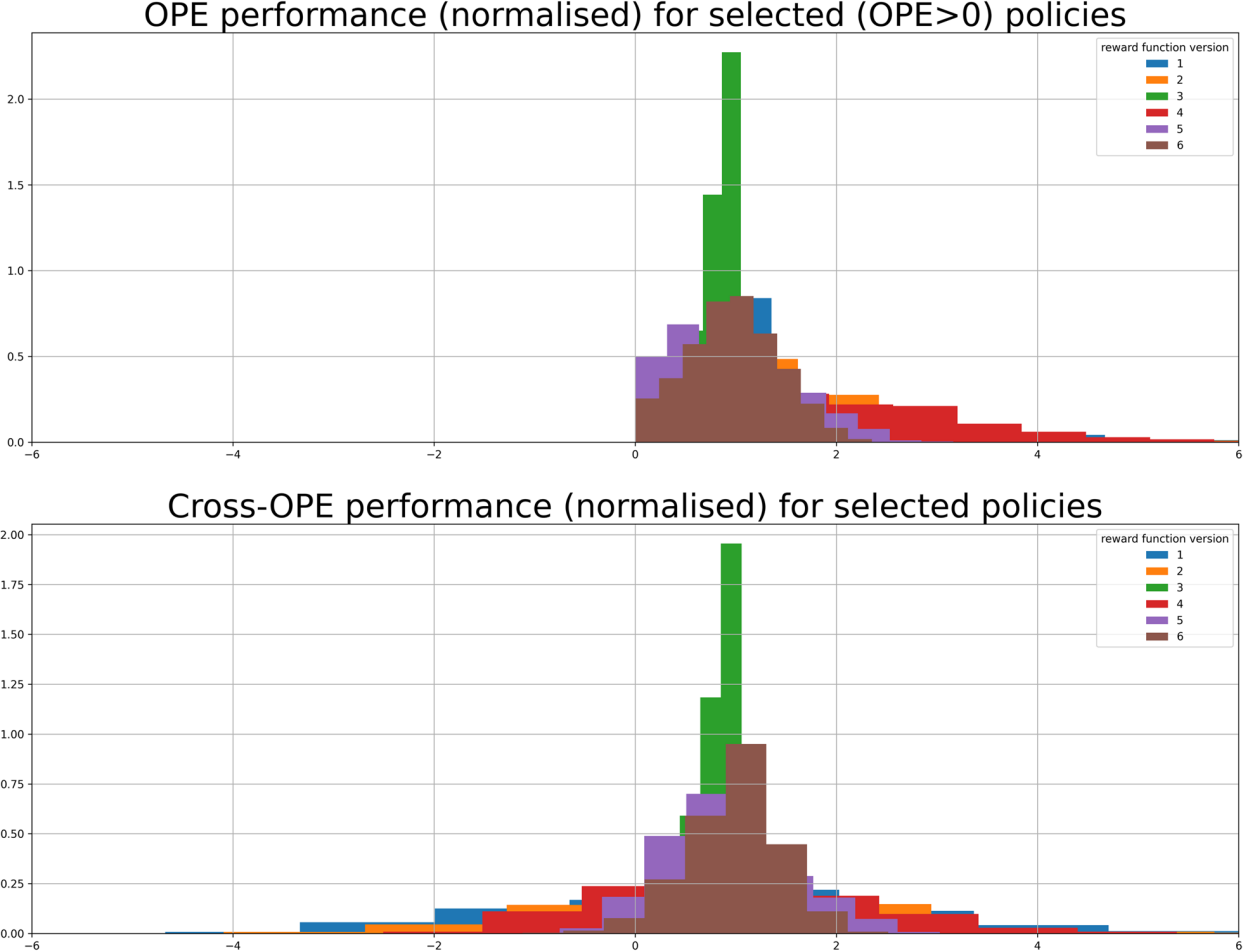
The histograms illustrate the evaluation outcomes with a detailing the Off-Policy Evaluation (OPE) for policies with positive OPE returns and b presenting the cross-OPE results. The vertical axis shows the density distribution, indicating data spread. The horizontal axis measures the relative performance or value of the target policy against the behaviour policy, using different reward functions without a specific unit. The "reward function version" corresponds to a series of weight factors: [0.25, 0.5, 1, 2, 4, 8], which are assigned to versions 1 through 6, respectively

For each model, we evaluated two policies, "greedy" and "king-knight", using the MAGIC OPE estimator. A total of 69,120 models were trained from which 138,240 policies were evaluated. From these, a set of 17,182 policies were selected and further evaluated using cross-OPE evaluation, as defined in the second evaluation round, see Fig. 3. Only 9.635 (56%) of cross-OPE evaluated models passed this evaluation phase. The results of this evaluation are shown in Fig. 4. Despite the successful performance of the models in the initial evaluation phase, a considerable number failed to exhibit comparable results during the cross-OPE evaluation utilising an alternate reward function shape. This may indicate the possibility of overfitting or the development of a policy that lacks generalisability. A final selection of top 100 models was based on cross-OPE performance and further explored in the clinical policy evaluation.

### Model evaluation

**Fig. 5:**
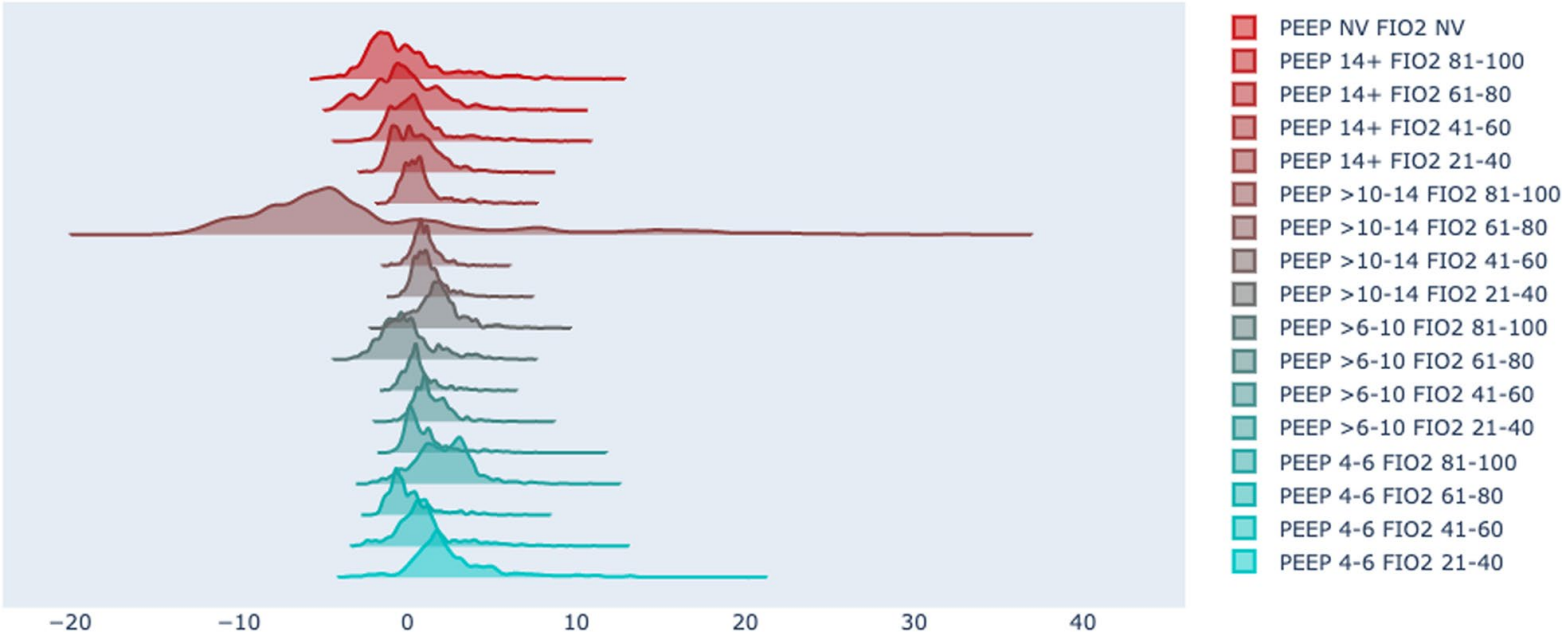
Distribution of Q-values for all state-action pairs within the historical dataset. The x-axis denotes the Q-values, and the y-axis represents their frequency. The plot reveals a pronounced shift to the left in the Q-value distribution for 'PEEP 10–14 and FiO2 80–100%', implying a lower expected reward. A policy based on this model will likely infrequently suggest 'PEEP 10–14 and FiO2 80–100%'. Each curve in the plot is labelled according to the corresponding 'PEEP' and 'FiO2' action pair, as specified in the legend

We used the Q-value distribution of the state-action pairs of the dataset to investigate how the model ranks different actions on average. Figure 5 provides an example of a model with policies that had good quantitative (top ten percentile and only positive OPE and cross-OPE results) results, but shows a bias against one specific action, 'PEEP 10–14 and FiO2 80–100%.' Although the policies show clinically reasonable action recommendations, the bias in this model could disqualify it from clinical implementation, highlighting that OPE results alone are insufficient for a robust model and policy evaluation.

### Policy evaluation

**Fig. 6:**
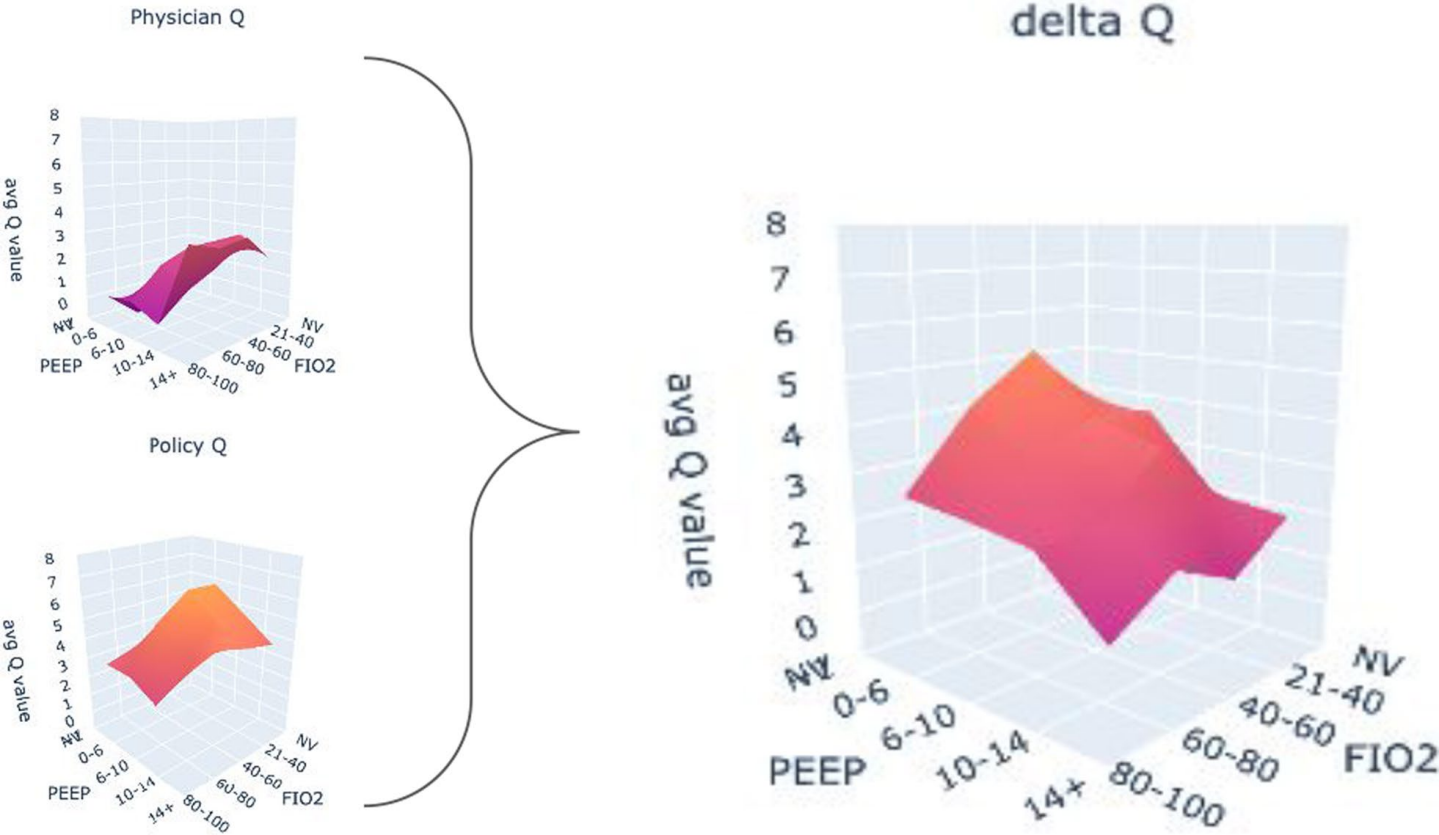
Displayed are three aggregate surface plots over the dataset: the first for physician-chosen actions, the second for policy-recommended actions, and the third, Delta-Q, which is calculated by subtracting physician Q-values from policy Q-values. The Delta-Q plot indicates smaller differences in high (14 +) PEEP and FIO2 (80–100%) settings, suggesting this action aligns closely with the policy's guidance. In contrast, larger Delta-Q values in lower PEEP and FIO2 ranges suggest greater divergence, indicating that these areas may have more room for optimisation in alignment with policy recommendations

Figure 6 presents the delta-Q surface plot for a policy applied across the dataset, highlighting areas needing optimisation. The delta-Q is lower for actions in the 'PEEP 14 + cmH20 and FiO2 80–100%' range, and higher in the lower PEEP (0–6 cmH20, 6–10 cmH20) and lower FiO2 (21–40%, 40–60%) ranges, indicating these as critical improvement areas. This trend is consistent across models that passed the OPE and cross-OPE evaluations, directing physicians to prioritise these ranges in mechanical ventilation strategies for COVID ARDS patients.

**Fig. 7:**
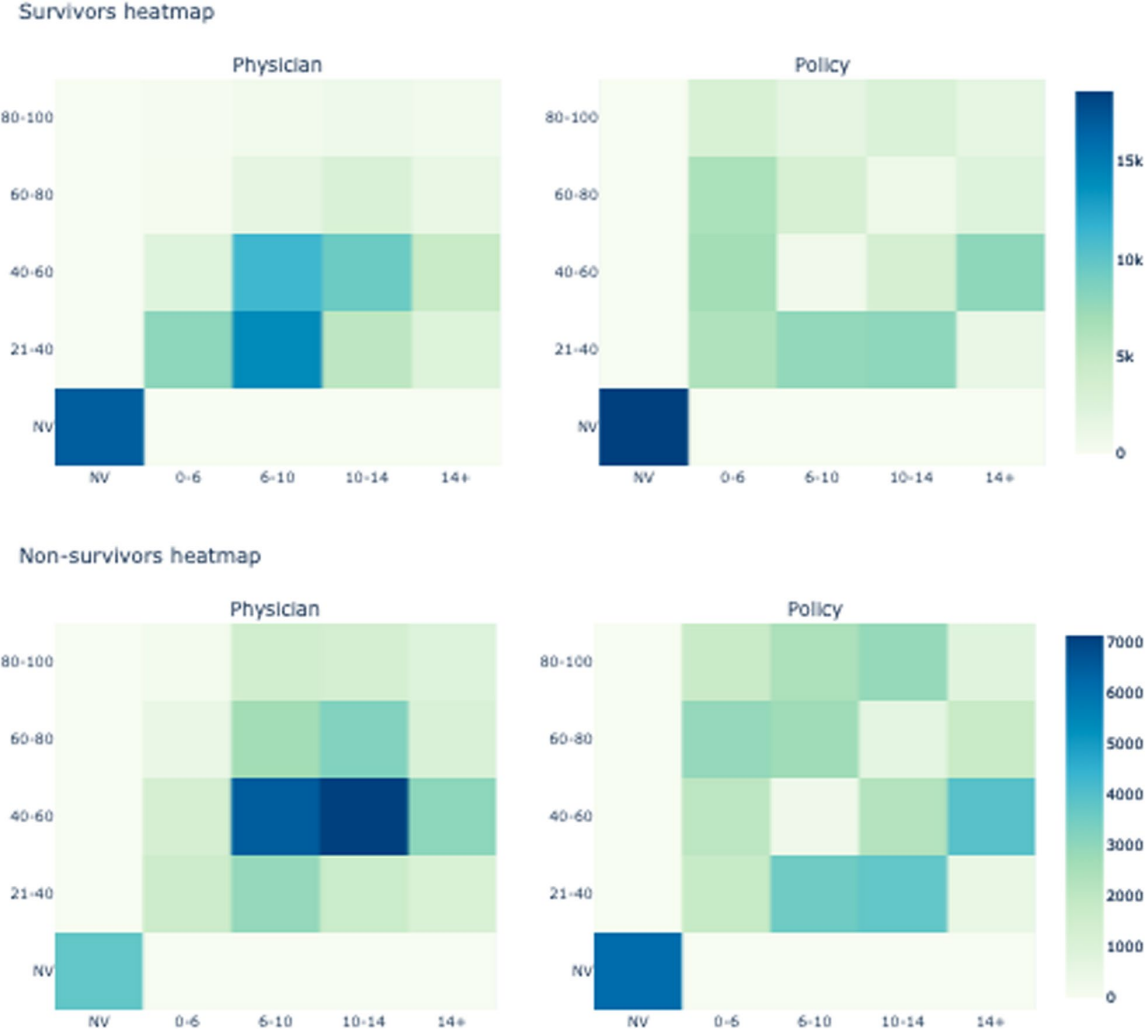
Comparison of action distributions between physician decisions and the optimal policy, segmented by patient outcome. The upper pair of heatmaps delineates the frequency of actions taken for survivors, contrasting actual physician choices with those suggested by the optimal policy. The lower pair of heatmaps mirrors this analysis for non-survivors. Across both sets, the x-axis categorises the level of PEEP and FiO2, while the y-axis sorts by FiO2 percentage. The NV label stands for non-invasively ventilated. The colour gradient represents the count of actions, with darker shades indicating higher frequencies

Inherent to our safety-restricted policy design, clinical decisions can be more optimal than the RL-derived policy, as the safety-restricted policy limits the selection to less aggressive choices, barring potentially optimal but riskier actions. This includes situations where transitioning from a lower PEEP and FiO2 setting to a significantly higher one cannot be advised by the safety-restricted policy. These instances where the AI and clinical policy differ significantly are spread across the action space, suggesting a nuanced understanding of policy performance and indicating a need for future research, particularly in time series analysis. Figure 7's action distribution analysis for subgroups further reveals that the optimal policy typically favours treatments with higher FiO2 and PEEP values, though not in combination, reflecting a complex balance in treatment decision-making.

### Clinical policy inspection and RL model application

**Fig. 8:**
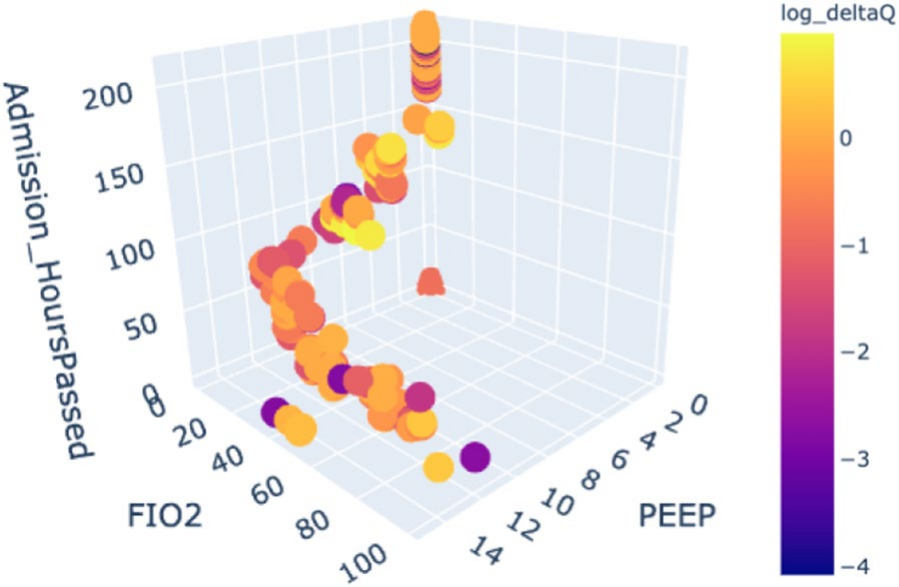
An example of a trajectory 3D visualisation of the two components of the action space, PEEP and FiO2 over time with log-Delta-Q values shown through varying hues

We introduce a method for applying RL to individual patient cases, exemplified in the log-Delta-Q trajectory 3D analysis, as presented in Fig. 8. This figure displays a 3D graph of PEEP, FiO2, and time, with log-Delta-Q values shown through varying hues. In specific instances, abrupt shifts in hue on the graph can signal physicians to reassess the patient's condition, suggesting potential changes in clinical state that might not be obvious through standard indicators. This method can assist physicians in prioritising their attention, aiding them in determining which patients may need more immediate and focused care.

## Discussion and conclusion

In this study, we proposed a method to assess RL model performance using cross-OPE, which allows for assessment of generalisability of trained policies. We also introduced a novel policy filter, "king-knight", that restricts model recommendations to actions similar to those taken by physicians. Additionally, we proposed to operationalise the application of RL in ICU settings by implementing delta-Q based clinical alerts. These alerts prompt physicians to reassess treatment plans, particularly PEEP/FiO2 settings, in response to changes in patient or ventilator parameters. Additionally, Fig. 8 provides a graphical representation to aid physicians in tracking patient progress, identifying unusual action choices, and exploring potential alternative treatment strategies. This approach may enhance real-time decision-making but also fosters a deeper understanding of patient-specific responses within the RL model's action space.

It is crucial to conduct a thorough clinical evaluation of trained RL models and policies before deployment in real-world settings. We showed how the use of off-policy evaluation alone may not be sufficient and may not identify models with clinically unexplainable behaviour. If a model's behaviour is not understandable or is at odds with clinical practice, it may not be trusted by physicians and could potentially lead to unsafe or suboptimal treatment decisions. Therefore, it is important to use a combination of methods, such as cross-OPE and clinical policy inspection, to ensure that the trained models and policies align with clinical expectations and have the desired performance.

It is important to note that there are limitations to these methods. One important caveat is that the proposed method relies heavily on domain expertise to define the components of the reward function and performing a clinical evaluation is labour intensive and time consuming. Nonetheless, to ensure the safe deployment of RL models in clinical practice, we believe that despite the added labour and time required this step is essential for the safe and effective deployment of RL models in the healthcare setting. Additionally, it should be mentioned that our novel approaches to model evaluation can mitigate but not completely solve challenges related to off-policy evaluation.

One limitation of this study is that it intentionally simplifies the action space, focusing primarily on optimising two key ventilatory settings. While this decision was made to make the model more efficient and interpretable, it raises the question of whether solely fine-tuning these parameters would significantly improve treatment outcomes without simultaneously optimising other crucial aspects of patient care, e.g. sedation, muscle blockers, haemodynamic stability or appropriate anticoagulation therapy. Additionally, it is important to note that this question may remain unanswered without empirical evidence, suggesting that an initial deployment of a simpler model may be practical, followed by iterative refinements to incorporate additional complexities as needed to enhance treatment efficacy.

Our proposed method also opens up new avenues for exploration, particularly in refining model selection through physician–patient consultations. Integrating patient preferences, like prioritising survival or quality of life, into the RL model's reward function is key to selecting a fitting reward function and corresponding optimal model, ensuring treatment advice aligns with each patient's specific preferences, thus enhancing patient-centric decision-making. The use of cross-OPE can assist researchers and physicians in creating models that perform well across a range of patient preferences. While there are limitations to these methods, they represent a step forward in the application of RL in healthcare, with the potential to improve treatment outcomes for critically ill patients.

Upcoming studies should focus on validating these methods across various clinical settings and adjusting RL models to align with evolving treatment standards. When planning for future localised applications of these models, it is also important to consider the centre-effect [35] variations in resources, practice patterns, and patient demographics may profoundly influence performance.

In conclusion, we have shown how cross-OPE can serve as a robust evaluation framework to identify policies with good generalisability. In addition, we demonstrated that policy restriction can help prevent potentially unsafe model recommendations. Finally, the novel delta-Q metric can be used to operationalise RL models in clinical practice. Our findings offer a promising pathway towards application of RL in intensive care medicine and beyond.

### Supplementary Information

**Additional file 1. **List of features.

**Additional file 2: **Reward function formulation for reinforcement learning model.

## Data Availability

All participating hospitals have access to the Dutch ICU Data Warehouse. External researchers can get access in collaboration with any of the participating hospitals. Contact details can be found on amsterdammedicaldatascience.nl. The code used for analysis is available upon request at gitlab.com.
