# Supplementary material for: Reinforcement learning for intensive care medicine: actionable clinical insights from novel approaches to reward shaping and off-policy model evaluation
Source: Intensive Care Med Exp. 2024 Mar 25;12:32. doi: 10.1186/s40635-024-00614-x (PMC10963714; doi:10.1186/s40635-024-00614-x)
Supplement: Supplementary file 2 — Additional file 2: Reward function formulation for reinforcement learning model. [file 40635_2024_614_MOESM2_ESM.docx]

**Additional file 1: Appendix SB: Reward Function Formulation for Reinforcement Learning Model**

In our proposed Reinforcement Learning (RL) framework, the reward function is a crucial component that dictates the learning process. The total reward R(s, a, s') for transitioning from state s to state s' due to action a is defined as a combination of intermediate and terminal rewards. Specifically, the reward function is formulated as follows:

R(s, a, s') = 𝓌 * R_intermediate(s, a) + 𝛧 * R_terminal(s')

where:

R_intermediate(s, a) represents the intermediate reward dependent on the current state and action.

R_terminal(s') is the terminal reward, which is only conferred at the final step of the finite horizon.

𝓌 is a weighting factor applied to the intermediate reward, introducing a tunable balance between the immediate and long-term outcomes. In our experiments, this weighting factor is varied across a set of predefined values: [0.25, 0.5, 1, 2, 4, 8].

𝛧 is an indicator function that evaluates to 1 if the current step is the last step of the finite horizon, and 0 otherwise.

This reward structure allows us to investigate the effects of emphasising short-term versus long-term rewards in the training of our DQN agent, providing insights into the optimal balance for effective decision-making in the context of our specific application domain.

Intermediate reward

The intermediate reward component is based on key clinical indicators: the PF-ratio and Dead Space Ventilation (DPV). The PF-ratio, representing the ratio of arterial partial pressure of oxygen (PaO2) to inspired oxygen concentration (FiO2), is a critical measure for assessing oxygenation levels and ARDS severity. Meanwhile, DPV, estimated using Enghoff's modification of Bohr's equation, reflects the efficiency of CO2 removal, a primary goal in mechanical ventilation. Our approach accounts for the variability and complexity of these parameters by focusing on relative changes, rather than fixed targets, to adaptively respond to the patient's dynamic condition. The following section delineates the specific conditions under which rewards and penalties are assigned in our intermediate reward function, emphasising the significance of relative changes in PF-ratio and DPV for invasively ventilated patients.

PF Ratio Conditions:

- If PF ratio > 300: Reward of +3.
- for PF ratio < 300:
  - If relative change in PF ratio > +20%: Penalty of -1.
  - if relative change in PF ratio < -20%: Reward of +1.
- for 150 < PF ratio ≤ 300 and invasively ventilated:
  - If -20% > relative change in PF ≤ 20%: Reward of +1
- for PF ratio < 150:
  - If -20% > relative change in PF ≤ 20%: Penalty of -1.

For invasively ventilated patients the following conditions also apply:

- If DPV > 3 mL/kg:
  - No relative change in DPV: Penalty of -2.
- If DPV ≤ 3 mL/kg:
  - Relative change in DPV > +20%: Reward of +1.
  - Relative change in DPV < -20%: Penalty of -1.

Conditions may overlap and the intermediate reward is the sum of all rewards and penalties for each state.

Terminal reward

The terminal reward in our Reinforcement Learning (RL) framework is determined based on a set of nested conditions related to patient outcomes following ICU admission. This component of the reward function reflects critical aspects of patient progress, including survival and discharge outcomes. The terminal reward is structured as follows:

- ICU Mortality Conditions:
- If death occurs within 72 hours: Penalty of -300.
- Else if death within 3 to 7 days: Penalty of -200.
- Else if death within 7 to 28 days: Penalty of -100.
- Else if death after 28 days: Penalty of -200.
- Hospital Mortality Conditions (Post-ICU Admission):
  - If death occurs within 72 hours: Penalty of -300.
  - Else if death within 3 to 7 days: Penalty of -150.
  - Else if death within 7 to 14 days: Penalty of -50.
  - Else if death within 14 to 28 days: Penalty of -10.
- ICU Discharge Speed (For Survivors after 28 days):
  - If discharged within 3 days: Reward of +150.
  - Else if discharged within 3 to 7 days: Reward of +100.
  - Else if discharged within 7 to 14 days: Reward of +50.
  - Else if discharged within 14 to 28 days: Reward of +50.
- Hospital Discharge Location (For Survivors):
- If discharged home: Reward of +150.
- Else if admitted from home and discharged to external care: Reward of +50.
- Else if admitted from and discharged to external care: Reward of +10.

Each of these conditions contributes to the total terminal reward for a patient's trajectory. The final reward for each patient is calculated by adding the values based on the specific conditions met from these categories, reflecting the overall outcome of the ICU stay and subsequent hospitalisation absent more exact measurements such as Quality-Adjusted Life Years. External care is defined as any healthcare facility such as a nursing home or a rehabilitation centre.
